# Long-term outcomes in antineutrophil cytoplasmic autoantibody–positive eosinophilic granulomatosis with polyangiitis patients with renal involvement: a retrospective study of 14 Chinese patients

**DOI:** 10.1186/s12882-016-0319-2

**Published:** 2016-07-26

**Authors:** Yinghua Chen, Yuemei Ding, Zhengzhao Liu, Haitao Zhang, Zhihong Liu, Weixin Hu

**Affiliations:** 1 National Clinical Research Center of Kidney Diseases, Jinling Hospital, Nanjing University School of Medicine, 305 East Zhongshan Road, Nanjing, 210002 Jiangsu China; 2 Jiangsu Jiangyin People’s Hospital, Jiangyin, China

**Keywords:** Eosinophilic granulomatosis with polyangiitis, Antineutrophil cytoplasmic autoantibody, Outcome

## Abstract

**Background:**

The clinic-pathological features and outcomes of Chinese patients with antineutrophil cytoplasmic autoantibody (ANCA)-positive eosinophilic granulomatosis with polyangiitis (EGPA) and renal involvement have not been studied.

**Methods:**

Fourteen EGPA patients with renal involvement were included. All patients underwent renal biopsy. Clinic-pathological features and outcomes were retrospectively analyzed.

**Results:**

The most common initial symptom of EGPA was asthma (57.1 %), followed by hemoptysis (21.4 %), gross hematuria (14.3 %), and arthritis (7.1 %). All patients had positive serum ANCA (anti-MPO in 12, anti-PR3 in 2). Elevated eosinophils (median 15 %, range 10–45 %) were found in all patients. The median serum IgE level was 463 g/L (range 200–1000 g/L). All patients presented with renal dysfunction, with a median SCr of 5.4 mg/dL (range 1.47–11 mg/dL), seven patients (50 %) required initial renal replacement therapy. Thirteen patients showed hematuria and proteinuria (median 1.1 g/24 h, range 0.5–7.8 g/24 h). Renal biopsy showed pauci-immune segmental necrotizing glomerulonephritis with crescents in 13 patients and acute interstitial nephritis in one patient. Twelve patients (85.7 %) showed renal interstitial eosinophil infiltration, among whom three had eosinophilic granuloma. Among seven patients (71.4 %) who required initial dialysis, 5 discontinued dialysis, one died, one received maintenance dialysis after glucocorticoids plus immunosuppressive for induction treatment. Twelve patients were followed up for a median of 43.5 months (range 6–83 months), during follow-up, two patients progressed to end-stage renal disease, nine had chronic kidney disease with eGFR < 60 mL/min, and two patients had normal eGFR.

**Conclusions:**

Renal involvement in ANCA-positive EGPA could be severe and showed varied renal histology. Although intensive immunosuppressive therapy effectively improved the renal function, the long-term renal survival was poor. Early diagnosis and treatment are essential to improve long-term renal survival.

## Background

Eosinophilic granulomatosis with polyangiitis (EGPA), previously termed Churg–Strauss syndrome [1], is a rare vasculitis disease, characterized by asthma, eosinophilia, and systemic vasculitis. EGPA is categorized as an antineutrophil cytoplasmic antibody (ANCA)-associated vasculitis (AAV), but only 35–40 % of EGPA patients are reported to be ANCA positive, mostly are MPO-ANCA positive [2, 3].

Renal disease has been reported in 25–45 % of EGPA [4, 5], with a higher incidence of renal involvement among ANCA-positive patients. Renal manifestations of the disease were diverse, most patients had mild urinary abnormalities [1, 6], whereas some presented with severe, rapidly progressive glomerulonephritis requiring dialysis [5]. Renal damage was reported to be more severe among ANCA-positive EGPA patients [5, 7]. The typical histopathological lesion was pauci-immune focal segmental necrotizing glomerulonephritis with crescent formation. However, other histological lesions have been reported, and eosinophilic interstitial infiltrates have been described [5]. The reported long-term renal survival of EGPA was variable. Most studies reported that renal function generally remained stable, whereas some studies reported that a proportion of patients progressed to ESRD, but all were reported European countries [8, 9]. In this study, we retrospectively analyzed the clinic-pathological features and outcomes of 14 Chinese patients with ANCA-positive EGPA.

## Methods

### Patients

Patients with EGPA were identified from AAV in the National Clinical Research Center of Kidney Diseases, Jinling Hospital, from January 1998 to December 2012. All patients met four or more of the following diagnostic criteria for EGPA, as proposed by the American Rheumatism Association in 1990 [10]: (1) asthma; (2) peripheral blood eosinophils > 10 %; (3) mononeuritis (including multiplex) or polyneuropathy; (4) nonfixed pulmonary infiltrates on roentgenography; (5) paranasal sinusitis; and (6) extravascular eosinophils proven histologically. All patients had renal involvement, and underwent renal biopsy.

### Clinical and laboratory data

Clinical measurement included age at disease onset, duration of EGPA, initial symptoms and organ involvement, red blood cell count of the urinary sediment, 24 h urinary protein excretion, and serum creatinine (SCr). Serum ANCA and anti-glomerular basement membrane antibody were detected by both indirect immunofluoresence and enzyme-linked immunosorbent assay. The estimated glomerular filtration rate (eGFR) was estimated by the Chronic Kidney Disease Epidemiology Collaboration equation [11].

Nephrotic syndrome was defined as proteinuria ≥ 3.5 g/24 h and serum albumin < 30 g/L. Rapidly progressive glomerulonephritis was defined as massive hematuria, ccompanied by a rapidly progressive decline in renal function. Chronic kidney disease (CKD) was defined as eGFR < 60 mL/min/1.73 m^2^ lasting for more than 3 months and stages of CKD were defined according to eGFR as Stage 3: eGFR 30–59 mL/min/1.73 m^2^, Stage 4: eGFR 15–29 mL/min/1.73 m^2^, or Stage 5 (ESRD): eGFR < 15 mL/min/1.73 m^2^ lasted for at least 3 months [12].

### Renal histology

Renal biopsy samples obtained from all patients were examined with light microscopy, immunofluorescence, and electronic microscopy. Renal tissues were routinely stained with hematoxylin and eosin, PAS, and PASM-Masson for light microscopy. Frozen sections were used for immunofluorescence. For electron microscopy, tissue samples were stained with sodium acetate and lead citrate and examined with a transmission electron microscope. Glomerular histology was categorized into four classes: focal (≥50 % normal glomeruli), crescentic (≥50 % glomeruli with cellular crescents), mixed (<50 % normal, <50 % crescentic, and <50 % globally sclerotic glomeruli), and sclerotic (≥50 % globally sclerotic glomeruli) [13]. Extensive foot process effacement was defined as an area of podocyte foot process effacement larger than 50 % of the capillary loop by electron microscopy.

### Treatment

Induction therapy included glucocorticoids, glucocorticoids plus mycophenolate mofetil (MMF,1–1.5 g/d), or intravenous cyclophosphamide pulse (IV-CTX) as previously reported [14]. For patients with severe renal damage or pulmonary hemorrhage received intravenous methylprednisolone (500 mg/d for 3–6d) pulse therapy and additional immunoadsorption, followed by oral prednisone (started at 0.6 mg/kg/d for 4 weeks, then reduced gradually to a maintenance dosage of 10 mg/d) [15].

Maintenance therapy included prednisone (10 mg/d) plus azathioprine (AZA,50–100 mg/d) or Tripterygium wilfordii polyglycoside (TW, a Chinese herbal medicine; 60 mg/d) [16].

### Statistical analysis

Data are presented as mean ± SD or median (range). Statistical analysis was performed with SPSS 16.0 statistical package (SPSS Inc., Chicago, IL, USA).

## Results

### Patient data

The 14 EGPA patients accounted for 5.0 % of the 282 Chinese AAV patients in our department during the study period. Eight were female and six were male, with a median age of 53 years (range 20–70 years), duration of EGPA 120 months (range 1–600 months) and duration of renal involvement 1.0 month (range 0.5–36 months). The duration between EGPA and renal involvement was 119 months (range 0–598 months) (Table 1). Prior to the admission, 12 patients received no glucocorticoids or immunosuppressive agents, two patients (Cases 4 and 9) received low-dose glucocorticoids.

**Table 1:** Clinic-pathological data of patients with EGPA and renal involvement

Case	Gender/Age range (years)	T1/T2 (months)	Onset symptom	Extra-renal involvement	Upro (g/24 h)	EOS (%)	IgE (g/l)	SCr (mg/dl)	Alb (g/l)	MPO-ANCA (RU/ml)	Renal class	Crescent (%)	GS (%)	Interstitial EOS	FPE
1	F/45-55	240/4	Asthma	N/NS	1.00	20	358	4.50	45.2	674.6	Mixed	39	49	+^b^	NA
2	F/25-35	24/24	Hemoptysis	L/A/SK/N	0.10	45	217	1.47	32.4	343.5	AIN	0	0	+	NA
3	F/18-25	1/1	GH	N	7.80	16	976	11.00	27	20.0	Crescentic	90	0	+	Extensive
4	F/55-65	600/28	Asthma	L/H/NS	0.97	31	1000	1.70	43.2	28.8	Mixed	40	27	+	NA
5	M/25-35	120/1	Asthma	L	1.14	10	316	1.68	41.7	86.3	Focal	19	25	−	Segmental
6	F/45-55	408/48	Asthma	L/SK	0.99	12	1000	5.90	34	161.0	Mixed	31	45	+	NA
7	M/65-75	120/1	Asthma	L	4.96	20	580	4.90	29	352.6	Crescentic	51	19	+	Extensive
8	F/55-65	2/1	Hemoptysis	L	0.50	10	200	10.50	34	180.0	Crescentic	80	0	+	Segmental
9	M/45-55	9/1	Arthritis	L/SK/A/N/E	0.53	21	287	9.40	37	156.4^a^	Mixed	9	41	−	NA
10	M/25-35	36/36	GH	N	12.30	14	1000	3.90	28.2	109.5^a^	Mixed	46	42	+	Extensive
11	M/55-65	240/1	Asthma	L/NS/N	0.50	20	567	6.09	29	101.5	Focal	16	16	+^b^	NA
12	F/45-55	600/2	Asthma	L/N	5.32	11	243	2.74	26.5	222.0	Mixed	23	30.8	−	NA
13	M/45-55	1/1	Hemoptysis	L/H	1.78	13	271	8.68	33.2	207.3	Sclerotic	10	80	+	Segmental
14	F/45-55	240/1	Asthma	L	4.45	14	567	6.50	25.3	384.8	Crescentic	79	21	+^b^	NA

*EGPA* eosinophilic granulomatosis with polyangiitis. *T1* total duration, *T2* duration of renal involvement, *EOS* eosinophilia, *GS* glomerular sclerosis, *FPE* foot process effacement, *GH* gross hematuria, *SK* skin, *E* eye, *N* sinusitis, *A* arthralgia, *NS* nervous system, *L* lung, *H* heart, *AIN* acute interstitial nephritis, ^a^ *PR3-ANCA*, ^b^eosinophilic granuloma, *NA* unavailable

### Laboratory measurements

All 14 patients were serum ANCA positive, twelve were P-/MPO-ANCA, with MPO-ANCA levels of 20–674.64 RU/ml, two were C-/PR3-ANCA.

All patients had elevated eosinophils in the peripheral blood (median 15 %, range 10–45 %) and elevated serum IgE level (median 463 g/L, range 200–1000 g/L). Eleven patients (78.6 %) presented with anemia (Table 1).

### Onset of symptoms

Eight patients (57.1 %) presented with asthma as the initial symptom, asthma were mostly paroxysmal, only one patient presented with persistent asthma. The median interval between the onset of asthma and systemic vasculitis was 239 months (range119–598 months). The remaining six patients initially presented with symptoms of systemic vasculitis, including hemoptysis (*n* = 3), gross hematuria (*n* = 2), and arthritis (*n* = 1) (Tables 1 and 3).

### Extra-renal manifestations

The lungs were the most common extra-renal organ involved, with pulmonary hemorrhage in six patients (42.9 %), interstitial lung disease in four (28.6 %), and pulmonary nodules in one (7.1 %). The upper respiratory tract was the second common involved. Other organ involvement included the skin, peripheral nerves, joints, ears, and eyes (Table 2).

**Table 2:** Extra-renal involvement in patients with EGPA

Extra-renal involvement	N (%)
General symptom, n (%)	6 (42.9 %)
Upper respiratory tract, n (%)	8 (57.1 %)
Lung, n (%)	11 (78.6 %)
Hemorrhage, n (%)	6 (42.9 %)
Interstitial pneumonia, n (%)	4 (28.6 %)
Nodules, n (%)	1 (7.1 %)
Skin purpura, n (%)	3 (21.4 %)
Peripheral neuropathy, n (%)	3 (21.4 %)
Arthragia, n (%)	2 (14.3 %)
Heart, n (%)	1 (7.1 %)
Iridocyclitis, n (%)	1 (7.1 %)

*EGPA* eosinophilic granulomatosis with polyangiitis

### Renal manifestations

Twelve patients (85.7 %) presented with rapidly progressive glomerulonephritis and two with CKD. All patients but one had both hematuria and proteinuria (Table 1). Urine protein ranged from 0.5–7.8 g/24 h (median 1.1 g/24 h). Five patients (35 %) presented with nephrotic syndrome, and two patients had gross hematuria. All patients had renal dysfunction, with a median SCr level of 5.4 mg/dl (range1.47–11.0 mg/dl) and a median eGFR of 9.8 mL/min/1.73 m^2^ (range 3.5–52.2 mL/min/1.73 m^2^), seven patients (50 %) initially required dialysis.

Thirteen renal biopsies showed pauci-immune segmental necrotizing glomerulonephritis, and one biopsy (Case 2) showed acute interstitial nephritis with normal glomeruli. Thirteen biopsies (92.8 %) showed glomerular crescents with the median crescents 39 % (range 9–90 %). Ten patients (71.4 %) showed glomerular segmental necrosis. The median glomerular sclerotic ratio was 26 % (range 0–80 %). Twelve patients (85.7 %) had renal interstitial eosinophilic infiltration, three of whom showed renal interstitial eosinophilic granuloma. Renal histologic classification included crescentic type in four, mixed in six, focal in two, and sclerotic in one. Six biopsies were evaluated with electron microscopy, and three of the patients with nephrotic syndrome showed extensive foot process effacement (Table 3).

**Table 3:** Treatment and follow-up of patients with EGPA

Case	Treatment		Follow-up (months)	Measurements at last visit				
	Induction	Maintenance		Hb (g/dl)	EOS (%)	Upro (g/24 h)	SCr (mg/dl)	MPO-ANCA (RU/ml)
1	MP/CTX (6g^a^)	Aza	8	11.4	5	0.50	1.58	320
2	MP/CTX (5.8 g)	Aza	83	13.4	6	0.10	0.56	0
3	MP/CTX (6 g)	Aza	63	13.0	2	2.04	1.21	0
4	Pred/CTX (6.4 g)	TW	26	13.7	2	1.02	2.46	0
5	Pred/MMF	Aza	28	12.1	2	0.75	2.19	31.8
6	IA/MP/MMF	Aza	56	11.1	3	0.14	2.26	63.3
7	IA/MP/MMF	Aza	41	11.4	0	1.86	2.41	21
8	IA/MP/MMF	TW	46	12.8	3	0.12	1.79	135
9	MP/MMF	TW	50	13.7	1	0.2	2.30	89.9
10	MP/MMF	Pred	6	6.6	14	0.72	MHD	82.5
11	MP/Pred	TW	47	14.0	2	0.45	1.83	0
12	MP/Pred	TW	12	12.6	0	1.83	2.40	156
13	IA/MP/Pred	Pred	5	10.0	1	1.38	MHD	133.7
14^b^	IA/MP	-	0	-	-	-	-	-

*EGPA* eosinophilic granulomatosis with polyangiitis, *MHD* maintenance hemodialysis *Pred* prednisone, *MP* methylprednisolone, *Pred* prednisone, *CTX* cyclophosphamide, *MMF* mycophenolate mofetil, *IA* immunoadsorption, *TW* Tripterygium wilfordii polyglycoside, *AZA* azathioprine. ^a^total dose of CTX, ^b^the patient died

### Treatment and outcomes

Induction treatment included glucocorticoids plus MMF regimen (*n* = 6) or IV-CTX regimen (*n* = 4), or glucocorticoids alone (*n* = 4; Cases 11 and 12 were complicated with pneumonia on admission, Case 13 was sclerotic type and case 14 died at 2 weeks of treatment). Twelve patients were given methylprednisolone pulse therapy; five patients received additional immunoadsorption therapy.

During induction period, five of seven patients (71.4 %) who needed initial dialysis were able to discontinue dialysis, three of them received the MMF regimen, one received the IV-CTX regimen and one received glucocorticoids alone. One patient (Case 13) required maintenance dialysis, and the other one (Case 14) died of cardiac arrhythmia at 2 weeks.

Maintenance therapy included glucocorticoids combined with AZA (*n* = 6), TW (*n* = 5) or glucocorticoids alone (*n* = 1) (Table 3). The other 12 patients were follow-up for a median of 43.5 months (range 6–83 months). At last visit, two patients (14.2 %) had normal renal function, nine (64.3 %) were CKD stage 3–4 with a mean SCr of 2.13 ± 0.03 mg/dl (range 1.58–2.45) and eGFR (20.3–39.8 mL/min/1.73 m^2^), and one patient (Case 10) progressed to ESRD.

None of the five patients who needed initial dialysis and were able to discontinue it progressed to ESRD during 46–63 months (mean 52.4 ± 7.0 months) follow-up. At last visit, the median SCr was 1.21–2.30 mg/dL with an eGFR of 24.7–64.4 mL/min/1.73 m^2^.

During the follow-up, serum ANCA turned negative in four patients, declined in eight patients. One patient (Case 2) relapsed with hemoptysis, skin rash, and eosinophilia, which were concurrent with increased serum ANCA level in the 25th month of follow-up. No patient died during maintenance treatment.

## Discussion

EGPA, the incidence was reported to be 0.5–6.8 patients per million per year [8, 9], accounted for 12.2–21.4 % of AAV in European countries [17, 18]. EGPA accounted for 5.0 % of patients with AAV in this study, which was consistent with a report from Japan, but was lower than that reported in European studies [19].

Typical EGPA presented with three stages [20]: the allergic stage (characterized by prodromal symptoms of asthma and anaphylactic rhinitis), the eosinophilic stage (marked by hypereosinophilia and eosinophilic infiltration in tissues), and the vasculitic stage (with systematic vasculitis). The patients in European studies were reported to have typical allergic disease; the incidence of asthma in those studies was as high as 96–100 % [9, 21]. Among our patients, only 57.1 % had a history of asthma or other allergic symptoms prior to the onset of vasculitis. The reported incidence of asthma among EGPA patients in Japan is 72.7–89 % [19, 22]. Hence, the reported incidence of asthma among patients with EGPA is lower in Asian populations than in European countries, a difference that might result from racial and geographic differences. In our study, vasculitis arose without prodromal allergic symptoms in 42.8 % of the patients, suggesting rapid disease progression. In fact, no significant association has been found between allergic disease and EGPA severity [21, 23, 24].

Previous studies [1, 6, 8] found that the renal involvement in EGPA is usually mild with a benign course, which is an important difference between EGPA and other types of AAV. Chumbley and colleagues [7] reported that six of 30 EGPA patients had isolated microscopic hematuria, and three had slightly increased SCr. Lanham et al. [8] reported that among 16 EGPA patients, only four had renal involvement. However, in recent years, several studies [2, 5, 25] found that EGPA patients with positive serum ANCA had severe renal damage. Sinico et al. [5] reported that among 30 EGPA patients who presented with rapidly progressive glomerulonephritis, 75 % were ANCA positive, and 50 % had renal dysfunction. Clutterbuck et al. [25] found that 87 % of EGPA patients had renal dysfunction and 18.6 % had nephrotic syndrome; four patients had SCr levels exceeding 500 μmol/L. Compared with previous reports, the renal damage of patients in our study was more severe; all patients had renal dysfunction and half required dialysis. Moreover, 35.7 % of patients had nephrotic syndrome, and the percentage of glomeruli with crescent lesions was high, with a mean of 38 %. It was unclear whether the difference in severity of renal involvement in our study versus others was associated with positive serum ANCA or with racial differences.

Lanham et al. [8] described the histological features of EGPA as eosinophilic tissue infiltration, extravascular granulomatosis, and necrotizing vasculitis [1, 6]. The former two histological findings were thought to differentiate EGPA from other types of AAV. However, it was reported that these specific lesions rarely existed simultaneously. In the present study, only three patients had all three of these lesions, but the proportion of eosinophilic infiltration and eosinophilic granuloma in the renal interstitium were higher in our study than in other reports [5, 25], consistent with increased eosinophils and IgE levels in the peripheral blood. This finding indicates that these pathological changes are not uncommon in the active stage of the disease, which is the key clue to the diagnosis of EGPA. We also found one patient who had only interstitial nephritis without glomerular involvement. Similar cases have been reported in the literature [5, 17, 26]. In addition, other renal diseases, such as IgA nephropathy [15] and focal segmental glomerulosclerosis [27], have also been reported, suggesting that the renal damage of EGPA can vary. Moreover, five patients had massive proteinuria, which was a higher percentage than that in previous reports [5, 25]. Electron microscopy showed extensive podocyte foot process fusion, which is characteristic of podocyte disease. Park et al. [27] reported the case of an EGPA patient with massive proteinuria, whose renal histology showed focal segmental glomerulosclerosis. This finding indicates that EGPA can also cause podocyte disease by undefined pathogenesis.

Immunosuppressive treatment could improve the renal function of EGPA [28, 29]. In this study, five of seven patients were able to discontinue dialysis. After immunosuppressive therapy with glucocorticoids combined with either MMF or IV-CTX, SCr levels returned to normal in one patient who needed initial renal replacement therapy. The currently recommended regimen is glucocorticoids combined with IV-CTX as induction therapy. However, the IV-CTX regimen had substantial adverse effects and relapses are frequent [30]. Although MYCYC [31], a randomized controlled study compared the efficacy of MMF and IV-CTX regimens, was unable to demonstrate that MMF was non-inferior to IV-CTX in induction therapy for AAV. Our previous study found that MMF was effective in inducing remission and improving renal function in Chinese patients with microscopic polyangiitis (MPA) [14], suggesting that MMF may have therapeutic potential in Chinese MPA patients. Other small sample, non-controlled studies also showed that MMF was effective in inducing remission in patients with AAV [32–35], indicating that glucocorticoids plus MMF might be an alternative regimen for the treatment of AAV. Because EGPA is a rare disease and no large-scale, randomized controlled trials for treatment. Despite the distinct differences in clinical character and pathogenesis among MPA, granulomatosis with polyangiitis and EGPA, the treatment regimens for these AAV are not different and the patients have been uniformly treated according to disease stage and severity. Both IV-CTX and MMF were effective in the treatment of EGPA in this study; however, given the small sample size of this study, the difference in treatment response between the two regimens was only observational.

During the follow-up period, only two patients had renal function return to normal; nine patients developed Stage 3–4CKD and two progressed to Stage 5 CKD. The long-term renal survival in this study was lower than that reported in other studies [5, 25, 36]. The study of the French Vasculitis Study Group demonstrated that SCr ≥140 μmol/L and proteinuria ≥1 g/d were risk factors for poor renal prognosis in EGPA. In the present study, 64.3 % of patients had both of the above risk factors. Moreover, the high proportion of glomerular crescents and sclerosis, severe renal interstitial fibrosis, and old age seen in our patients were all considered to be poor renal prognosis [28, 29]. In addition, IMPROVE study [37] found that MMF was less effective than AZA for maintaining remission and glucocorticoids combined with AZA has been recommended as the maintenance therapy for AAV. But in this study, only half of the patients were given AZA as maintenance therapy, the dosage of AZA was also lower than that recommended by EULAR [38], which may be associated with the increased risk of renal relapse and low renal survival rate.

This study has several limitations. First, all patients were Chinese, so the results of this study might not apply to other populations. Second, all patients were ANCA positive, we did not compare the difference between ANCA-positive and ANCA-negative EGPA. Third, sample size was small and the conclusions need further investigation. Forth, for a retrospective study, the treatment regimens and the dosage of the immunosuppressive agents were not uniform. These factors may affect our assessments of the clinical features, renal outcomes and treatment efficacy for patients with EGPA.

## Conclusions

Renal involvement in ANCA-positive Chinese EGPA patients could be severe, and long-term renal survival was poor in this study. Intensive immunosuppressive therapy effectively improves renal function, therefore, early diagnosis and treatment are essential to improve long-term renal survival.

## Abbreviations

AAV, ANCA associated vasculitis; ANCA, antineutrophil cytoplasmic autoantibody; AZA, azathioprine; CKD, chronic kidney disease; eGFR, estimated glomerular filtration rate; EGPA, eosinophilic granulomatosis with polyangiitis; IV-CTX, intravenous cyclophosphamide pulse; MMF, mycophenolate mofetil; SCr, serum creatinine; TW, tripterygium wilfordii polyglycoside
